# Triggered Immune Response Induced by Antigenic Epitopes Covalently Linked with Immunoadjuvant-Pulsed Dendritic Cells as a Promising Cancer Vaccine

**DOI:** 10.1155/2020/3965061

**Published:** 2020-04-04

**Authors:** Chumeng Chen, Mohanad Aldarouish, Qilong Li, Xiangzhen Liu, Feng Han, Hui Liu, Qijun Qian

**Affiliations:** ^1^Xinyuan Institute of Medicine and Biotechnology, College of Life Sciences and Medicine, Zhejiang Sci-Tech University, Hangzhou 310018, China; ^2^Shanghai Cell Therapy Research Institute, Shanghai 201805, China; ^3^Shanghai Research and Development Center, Shanghai Cell Therapy Group, Shanghai 201805, China; ^4^Immune Cell Division, Shanghai Cell Therapy Group, Shanghai 201805, China

## Abstract

The success of peptide-based dendritic cell (DC) cancer vaccines mainly depends on the utilized peptides and selection of an appropriate adjuvant. Herein, we aimed to evoke a broad immune response against multiple epitopes concurrently in the presence of immunoadjuvant. Three synthetic HLA-A∗0201-restricted peptides were separately linked with HMGB1-derived peptide (SAFFLFCSE, denoted as HB_100-108_) as immunoadjuvant via double arginine (RR) linker and loaded onto human monocyte-derived DCs. Peptide uptake was detected by immunofluorescence microscopy and flow cytometry. The maturation and activation status of pulsed DCs were monitored by detection of the expression of specific markers and released cytokines. The ability of peptide-pulsed DCs to activate allogeneic T cells has been assessed by a degranulation assay and detection of secreted cytokines. The lytic activity of effector T cells against cancer cells in vitro was analyzed by a lactate dehydrogenase (LDH) assay. Results revealed that DCs efficiently take up peptides+HB_100-108_ and expressed higher levels of surface markers (HLA-ABC, HLA-DR, CD80, CD86, CD83, CD40, and CCR7) and proinflammatory cytokines (IL-6, IFN-*γ*, TNF-*α*, and IL-12) than control DCs, free peptide-pulsed DCs, and free HB_100-108_-pulsed DC groups. Moreover, peptides+HB_100-108_/pulsed DCs were capable of activating allogeneic T cells and enhance their lytic activity against a pancreatic cancer cell line (PANC-1) in vitro. These findings suggest that antigenic peptides covalently linked with HB_100-108_/pulsed DCs could be a promising strategy to improve the current DC-based cancer vaccines.

## 1. Introduction

The unique characteristics of dendritic cells (DCs) as the most potent antigen-presenting cells (APCs) lead them to be considered as a promising tool for cancer immunotherapy [1]. In the last few years, there has been a growing interest in peptide-, RNA-, and DNA-based DC vaccines as an effective approach for cancer immunotherapy due to its promising results in achieving significance and durable treatment responses with mild adverse events [2, 3]. Peptide-based vaccines provide several advantages in comparison to other types of cancer vaccines. They are easily synthesized, stable in many storage conditions, and safe, and they could enhance an effective CD4 and CD8 immune response. However, the success of the peptide-based vaccine strategy mainly depends on the utilized peptides and selection of an appropriate adjuvant [4].

High mobility group box 1 (HMGB1) is highly conserved protein in mammals that translocates to the nucleus to regulate the gene expression and released during cell injury and inflammation [5]. HMGB1 is composed of two DNA-binding motifs, box A and box B, in addition to C tail [6]. It has been found that HP91, a short peptide corresponding to amino acids 91–108 in the B box, has the ability to enhance the maturation and activation of DCs, induce the secretion of proinflammatory cytokines such as IL-6 and IL-12, and trigger the polarization of Th1 cells [7–9]. Saenz and his group demonstrated that HP91 could function as adjuvant in vivo through potentiating cellular and humoral immune responses [10]. Further studies showed that the region of HP91 which is responsible for its immunostimulatory function is located in the C-terminal, a peptide corresponding to amino acids 100-108 of HMGB1 [10, 11]. In this study, we denoted this short peptide as HB_100-108_.

It is well known that no single antigenic peptide might be adequate to achieve an efficient antitumor immune response. Thus, multiple epitopes are required to elicit a broad immune response [12]. Consequently, combining multiple tumor-associated antigens (TAA) could be a preferable strategy to elicit strong antitumor immunity.

A large number of studies confirmed that the following tumor-associated antigens, survivin, human epidermal growth factor receptor 2 (Her2), and carcinoembryonic antigen (CEA), are overexpressed in a variety of tumors, by which survivin is overexpressed in the lung, breast, pancreatic, and melanoma [13–16]; Her2 is overexpressed in the breast, stomach, ovary, uterine serous endometrial carcinoma, colon, pancreatic, bladder, lung, uterine cervix, head and neck, and esophagus cancer [17, 18]; and CEA is overexpressed in gastric, colorectal, breast, ovarian, lung, and pancreatic cancer [19].

In this study, human monocyte-derived DCs (moDCs) were pulsed with three antigenic peptides, survivin, Her2, and CEA, which are covalently linked with HB_100-108_ via protease-sensitive linker (double arginine (RR)). Results revealed that peptides covalently linked with HB_100-108_ (denoted as peptides+HB_100-108_) were efficiently taken up by immature DCs and significantly induced their maturation and activation compared with free peptides without HB_100-108_ (denoted as peptides-HB_100-108_). Moreover, peptides+HB_100-108_/pulsed DCs greatly induced the activation and lytic activity of allogeneic T cells by which they exhibited a potent cytotoxicity against tumor cells in vitro.

## 2. Materials and Methods

### 2.1. DC Generation

Monocyte-derived DCs were generated from peripheral blood monocyte (PBMC) by standard Ficoll density centrifugation (GE Healthcare, Uppsala, Sweden) to isolate PBMCs from patient leukapheresis samples. PBMCs were plated in serum-free AIM-V media (Life Technologies, Grand Island, NY) and allowed to adhere to 0.22 *μ*m filter-capped culture flasks (TPP, Germany). After 2 hours, the nonadherent cells were removed, and adherent monocytes were subsequently cultured for 6 days in AIM-V containing 50 ng/ml rhIL-4 (R&D Systems, Minneapolis, MN) and 100 ng/ml rhGM-CSF (Sanofi, Bridgewater, NJ). On day 3, half of the medium was replaced with fresh medium containing GM-CSF and IL-4. In some experiments, a maturation cocktail containing 100 IU/ml IFN-*γ*, 30 *μ*g/ml poly(I:C), and 5 *μ*g/ml R848 was used to mature the generated DCs.

### 2.2. Synthetic Peptides

The following synthetic HLA-A∗0201-restricted peptides, survivin (LTLGEFLKL), Her2 (RLLQETELV), CEA (YLSGANLNL), and HB_100-108_ (SAFFLFCSE), were synthesized with purity greater than 95%. Following their synthesis, the first three peptides were linked with HB_100-108_ via a protease-sensitive linker (double arginine (RR) residue sequence). It is expected that once DCs internalize a peptide-RR-HB_100-108_ construct, the intracellular proteases would cleave at the RR site and separate peptide from HB_100-108_ and thus could enhance the processing and presentation of engulfed peptides [20]. Peptides were purchased from Shanghai Top-peptide Bio Co., Ltd. (Shanghai, China). To examine their phagocytosis by DCs, peptides±HB_100-108_ were labeled with FITC fluorochrome at their N-terminus. All peptides were dissolved in PBS.

### 2.3. Peptide Uptake Assay

Immature moDCs, generated as described above, were seeded in a 24-well plate and pulsed separately with synthetic peptides±HB_100-108_-coupled FITC (40 *μ*g/ml for each peptide) for 1 hour at 37°C, 5% CO_2_. After a triple wash with PBS, cells were examined by fluorescent microscopy and the uptake was quantified by FACS analysis. To quench the extracellular FITC signal, 50 *μ*g/ml of trypan blue was added to the cell suspension prior to flow cytometry analysis. Unpulsed DCs were used as the negative control. Cells that were found positive for FITC were considered as cells that had successfully engulfed peptides.

### 2.4. Flow Cytometry

The purity of generated DCs was assessed as CD14^−^CD11C^+^ by staining 1 × 10^6^ cells with anti-CD11C-APC and anti-CD14-FITC (BD Biosciences, San Diego, CA, USA).

Immature moDCs were resuspended to 1 × 10^6^ cells/ml and loaded with peptides±HB_100-108_ (40 *μ*g/ml for each peptide) or 40 *μ*g/ml of free HB_100-108_ at 37°C, 5% CO_2_. After one hour, pulsed DCs were washed and cultured overnight. The following monoclonal antibodies (mAbs) were used to characterize the maturation and activation status of DCs: anti-HLA-ABC-PE, anti-HLA-DR-PE/Cy7, anti-CD80-PE, anti-CD86-APC, anti-CD83-APC, anti-CD40-Alexa Fluor 700, and anti-CCR7-PE/Cy7 (BioLegend, San Diego, CA, USA). Isotype-matched fluorescent antibodies were used as negative controls. Cells were incubated with antibodies at 4°C for 30 minutes. After washing, samples were detected by a FACSCalibur analyzer (BD Biosciences, San Jose, CA, USA). The data were analyzed by FlowJo software (TreeStar).

### 2.5. Enzyme-Linked Immunosorbent Assay (ELISA)

Immature moDCs were resuspended to 1 × 10^6^ cells/ml and loaded with peptides±HB_100-108_ (40 *μ*g/ml for each peptide) or only 40 *μ*g/ml of free HB_100-108_ at 37°C, 5% CO_2_. After one hour, pulsed DCs were washed and cultured for 48 hours. The secreted IL-12 was detected by a IL-12 ELISA kit (Sino Biological, China) according to the manufacturer's protocol. The optical density (OD) of samples was assessed at 550 nm using a microtiter plate spectrophotometer (Beckman Coulter detection platform, USA).

### 2.6. Degranulation Assay and Cytokine Detection

Peptides±HB_100-108_/pulsed DCs were cultured in the presence of a maturation cocktail (100 IU/ml IFN-*γ*, 30 *μ*g/ml poly(I:C), and 5 *μ*g/ml R848) for 24 hours. Cells were washed extensively and seeded with responder allogeneic T cells at a DC : *T* cell ratio of 1 : 10 for 18 hours. Supernatants were collected at the end of culture, and cytokine production was detected using a cytometric bead array (CBA) kit (BD Biosciences), following the manufacturer's instructions.

For the CD107a degranulation assay, allogeneic T cells were stimulated with empty DCs or peptides±HB_100-108_/pulsed DCs (at a DC : *T* cell ratio of 1 : 10) in the presence of GolgiStop (monensin, BD) and anti-CD107a-APC mAb (BD Pharmingen). After incubation for 12 hours at 37°C, cells were collected and stained with anti-CD8-PE mAb (BD Pharmingen) and analyzed by flow cytometry.

### 2.7. Cancer Cells

The human pancreatic cancer PANC-1 cell line (ATCC® CRL-1469™) was cultured in DMEM supplemented with 10% FBS and penicillin/streptomycin at 37°C in a humidified 5% CO_2_ atmosphere.

### 2.8. Cytotoxicity Assays

Peptides±HB_100-108_-pulsed DCs were matured in the presence of a maturation cocktail, followed by coculturing with allogeneic T cells at DC : *T* cell ratios of 1 : 10 for 24 hours. Then, T cells were collected as effector cells, and Panc-1 cells were used as the target cells. Effector cells included the negative control group (T cells without precoculturing with DCs), empty DC group (T cells stimulated with nonpulsed DCs), peptides-HB_100-108_ group (T cells stimulated with free peptides/pulsed DCs), and peptides+HB_100-108_ group (T cells stimulated with peptides covalently linked with HB_100-108_/pulsed DCs). Effector cells and target cells (PANC-1 cancer cell line) were incubated at ratios of 5 : 1 for 4 h at 37°C in 96-well plates. The activity of T cells against the target tumor cells was measured by an LDH cytotoxicity assay kit (Beyotime, China) following the manufacturer's instructions. The cytotoxicity of the T cells was calculated as a percentage of specific lysis using the following formula: %specific lysis = (effector/target release − spontaneous release)/(maximal release − spontaneous release) × 100%. Data are presented as the means ± standard deviation.

### 2.9. Statistical Analysis

Statistical analyses were carried out using GraphPad Prism 5.0 (GraphPad Software, San Diego, CA). The mean ± SD was determined for each treatment group in the individual experiments. Differences among groups were analyzed using Student's *t*-test, and *P* < 0.05 was considered statistically significant.

## 3. Results

### 3.1. Immature moDCs Efficiently Take Up Peptides Covalently Linked with HB_100-108_

**Figure 1:**
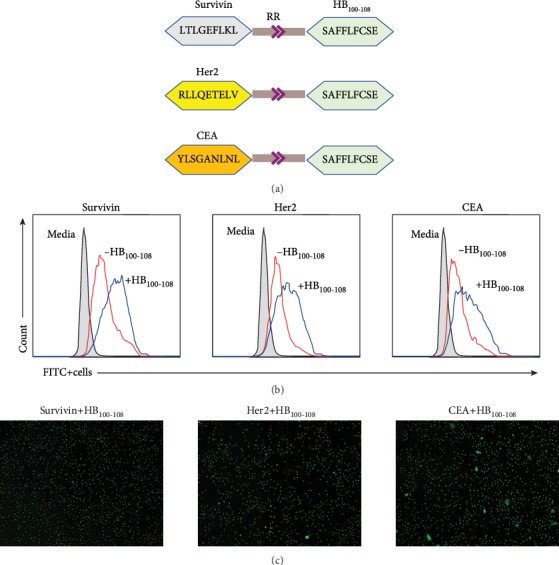
Immature DCs efficiently phagocytized antigenic peptides that are covalently linked with HB_100-108_. (a) A schematic diagram illustrates the synthetic peptides used in this study. Three HLA-A∗0201-restricted peptides (survivin, Her2, and CEA) were covalently linked with HB_100-108_ via double arginine (RR) residues as a protease-sensitive linker. (b) Immature moDCs were pulsed with 40 *μ*g/ml of FITC fluorochrome-conjugated peptides±HB_100-108_ for 1 hour at 37°C. After a triple wash with PBS, the uptake was quantified by FACS analysis. To quench the extracellular FITC signal, 50 *μ*g/ml of trypan blue was added to the cell suspension prior to flow cytometry analysis. Unpulsed DCs were used as the negative control. (c) Immunofluorescence microscopy images showing the uptake of FITC-peptides+HB_100-108_ by immature moDCs.

In this study, three antigenic synthetic peptides (survivin, Her2, and CEA) were covalently linked with HB_100-108_ (as immunoadjuvant) via double arginine (RR) residues as a protease-sensitive linker (Figure 1(a)). To detect whether the covalent linking of synthetic peptides with HB_100-108_ via RR linker could accelerate their acquisition by DCs, cells were incubated with survivin±HB_100-108_, Her2±HB_100-108_, or CEA±HB_100-108_ in culture medium for 1 hour at 37°C. All peptides were conjugated with FITC. Flow cytometry results showed that DCs more efficiently take up peptides covalently linked with HB_100-108_ than single free peptides (Figure 1(b)). The high efficiency of DCs to engulf peptides+HB_100-108_ was also confirmed by immunofluorescence microscopy (Figure 1(c)). These findings indicate that HB_100-108_ could play an important role in the acceleration of peptide phagocytosis by immature DCs.

### 3.2. Maturation and Activation of DCs Were Induced by Peptides Covalently Linked with HB_100-108_

To determine the optimum concentration of peptides covalently linked with HB_100-108_ that promote the highest level of DC maturation and activation, cells were loaded with different concentrations of peptides+HB_100-108_ (0, 20, 40, and 60 *μ*g/ml for each peptide) for 1 hour. Then, cells were collected, washed, and cultured overnight followed by detection of surface markers by flow cytometry. Data revealed that peptides+HB_100-108_ induced the expression levels of HLA-ABC, HLA-DR, CD80, CD83, CD40, and CCR7 in a dose-dependent manner without a significant difference between 40 and 60 *μ*g/ml (Supplementary Figure 1). Based on these findings, 40 *μ*g/ml of peptides+HB_100-108_ was used as a preferable concentration in the next experiments.

**Figure 2:**
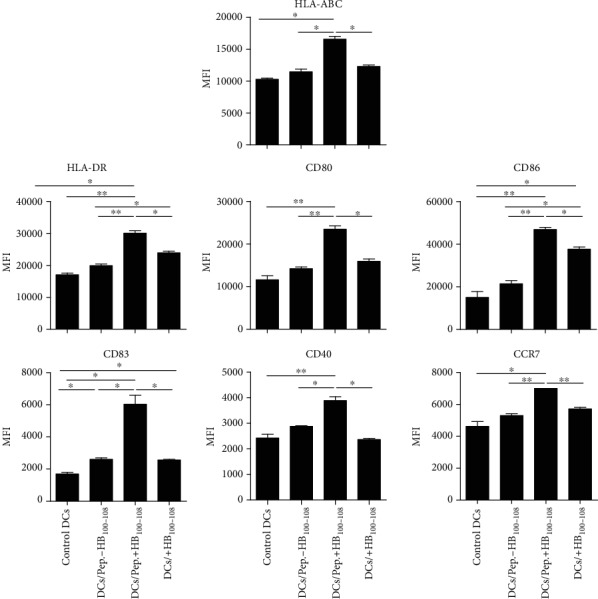
Peptides+HB_100-108_ enhanced the maturation and activation of immature DCs. Immature moDCs were left untreated or pulsed with peptides±HB_100-108_ or free HB_100-108_ (each with 40 *μ*g/ml) for 1 hour at 37°C. Then, cells were washed and cultured overnight. The expression of HLA-ABC, HLA-DR, CD80, CD86, CD83, CD40, and CCR7 was measured by flow cytometry. Results represent the mean ± SEM of three independent experiments.

To detect whether peptides+HB_100-108_ could induce the maturation and activation of DCs, immature DCs were divided into four groups: group (1) cells left untreated; group (2) cells incubated with free peptides: survivin, Her2, and CEA; group (3) cells incubated with survivin+HB_100-108_, Her2+HB_100-108_, and CEA+HB_100-108_; and group (4) cells incubated with free HB_100-108_. After 1 hour, cells were collected, washed, and cultured overnight followed by detection of surface markers by flow cytometry. Results showed that peptides+HB_100-108_/pulsed DCs exhibited remarkably increased expression of HLA-ABC, HLA-DR, CD80, CD86, CD83, CD40, and CCR7 compared to the other three groups. Importantly, free HB_100-108_ failed to upregulate the expression of those markers on pulsed DCs (except HLA-DR and CD86) compared with the control and peptides-HB_100-108_/pulsed DC groups (Figure 2). These data suggest that coupling of antigenic peptides with HB_100-108_ could serve as an efficient strategy to enhance the maturation and activation of DCs.

### 3.3. Peptides Covalently Linked with HB_100-108_/Pulsed DCs Secreted Abundant Levels of Proinflammatory Cytokines

**Figure 3:**
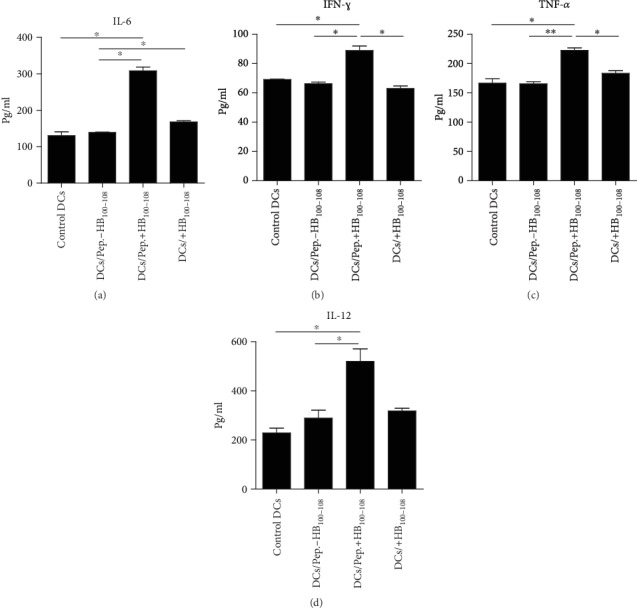
Peptides+HB_100-108_/pulsed DCs secreted abundant levels of proinflammatory cytokines. Immature moDCs were left untreated or pulsed with peptides±HB_100-108_ or free HB_100-108_ (each with 40 *μ*g/ml) for 1 hour at 37°C. Then, cells were washed and cultured for 24 hours, followed by detection of the secreted IL-6 (a), IFN-*γ* (b), and TNF-*α* (c) in the culture media by a cytometric bead array (CBA) kit. (d) IL-12 was detected by ELISA after 48 hours. Results represent the mean ± SEM of three independent experiments.

To investigate the ability of peptides covalently linked with HB_100-108_ to enhance the secretion of proinflammatory cytokines by DCs, cells were divided into four groups and treated as mentioned in the previous section. After 1 hour of incubation, cells were collected, washed, and cultured for 48 hours to detect IL-12 by ELISA and for 24 hours to detect other cytokines. Supernatants were collected, and the level of IL-12 was detected by a CBA kit using flow cytometry. Data revealed that peptides+HB_100-108_/pulsed DCs secreted abundant levels of IL-6 (Figure 3(a)), IFN-*γ* (Figure 3(b)), TNF-*α* (Figure 3(c)), and IL-12 (Figure 3(d)) compared to the other three groups. Consistent with the results of surface markers, the levels of those cytokines were comparable between the control DCs, peptides-HB_100-108_, and HB_100-108_ groups. These results demonstrate that peptides+HB_100-108_ have a considerable potential to promote DCs to secrete proinflammatory cytokines, by which they are important in skewing of T cell responses.

### 3.4. Peptides Linked with HB_100-108_/Pulsed DCs Elicited Allogeneic T Cells to Produce Several Cytokines and Induced the Lytic Activity of CD8^+^ T Cells

**Figure 4:**
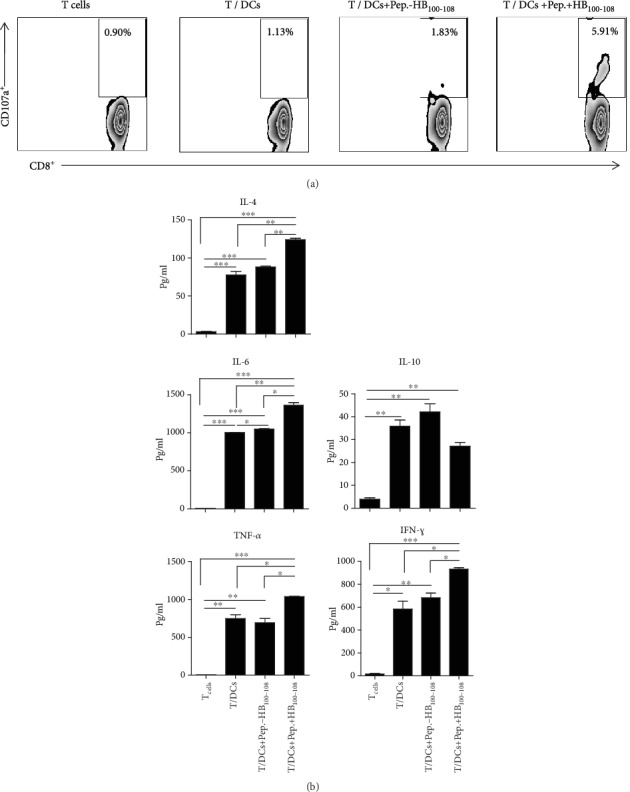
Peptides+HB_100-108_/pulsed DCs induced the degranulation of CD8^+^ T cells and enhanced allogeneic T cells to secrete inflammatory cytokines. Immature moDCs were left untreated or pulsed with peptides±HB_100-108_ (each with 40 *μ*g/ml). Cells were washed and matured in the presence of a maturation cocktail for 24 hours. (a) Allogeneic T cells were stimulated with empty DCs or peptides±HB_100-108_/pulsed DCs at *T* : DC cell ratio of 5 : 1 in the presence of GolgiStop and anti-CD107a-APC mAb. After incubation for 12 hours at 37°C, cells were collected and stained with anti-CD8-PE mAb and analyzed by flow cytometry. (b) Empty DCs or peptides±HB_100-108_/pulsed DCs were cocultured with responder allogeneic T cells at a DC : *T* cell ratio of 1 : 5 for 18 hours. Supernatants were collected at the end of culture, and cytokine production was detected using a cytometric bead array (CBA) kit. Results represent the mean ± SEM of three independent experiments.

To investigate the lytic activity of CD8^+^ T cells, allogeneic T cells were cocultured with empty DCs or peptides±HB_100-108_/pulsed DCs for 12 hours in the presence of anti-CD107a-APC mAb; then, cells were stained with anti-CD8-PE mAb and analyzed by flow cytometry. As shown in Figure 4(a), only peptides+HB_100-108_/pulsed DCs were able to induce a robust degranulation of CD8^+^ T cells, as identified by the expression of surface marker CD107a, indicating that those T cells could secrete cytotoxic effector molecules upon encountering DC-loaded peptides+HB_100-108_, whereas empty DCs and DC-loaded peptides-HB_100-108_ failed to enhance the degranulation of CD8^+^ T cells.

To evaluate the effect of DC-loaded peptides on the cytokine secretion by T lymphocytes, allogeneic T cells were cultured alone as a negative control (group 1) or cocultured with empty DCs (group 2), peptides-HB_100-108_/pulsed DCs (group 3), or peptides+HB_100-108_/pulsed DCs (group 4) at DC : *T* cell ratios of 1 : 10. 18 hours later, secreted cytokines were detected in the supernatant by the CBA kit. Data showed that the levels of secreted IL-4, IL-6, TNF-*α*, and IFN-*γ* in group 4 (peptides+HB_100-108_/pulsed DCs/T cells) were significantly higher than those of the other three groups. Importantly, the levels of secreted IL-10 in group 4 were obviously lower than those in group 2 (empty DCs/T cells) and group 3 (peptides-HB_100-108_/pulsed DCs/T cells) (Figure 4(b)). Collectively, these results suggest that peptides covalently linked with HB_100-108_/pulsed DCs are potent to promote the lytic activity of T cells, as well as enhance the secretion of type 1 and type 2 cytokines and diminish the secretion of the inhibitory cytokine IL-10.

### 3.5. Peptides Linked with HB_100-108_/Pulsed DCs Induced the Cytolytic Activity of T Cells against PANC-1 Cell Line In Vitro

**Figure 5:**
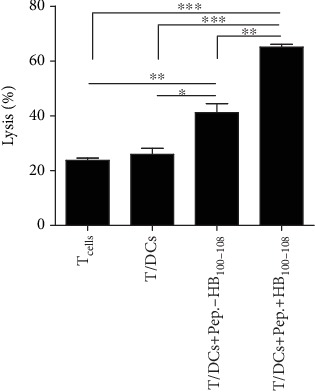
T cells stimulated by peptides+HB_100-108_/pulsed DCs exert considerable lysis of the PANC-1 cell line in vitro. After stimulation with empty DCs or peptides±HB_100-108_/pulsed DCs for 24 hours or no stimulation, allogeneic T cells were collected as the effector cells and the PANC-1 cancer cell line was used as target cells. The target cells were cocultured with the effector cells at a *T* : *E* ratio of 1 : 5. The cytolytic activity was assessed by measurement of lactate dehydrogenase (LDH) release. This experiment has been performed in triplicate and repeated three times with similar results.

It has been documented that pancreatic tumor cells expressed high levels of survivin, Her2, and CEA antigenic peptides [21]. To test whether the peptides±HB_100-108_/pulsed DCs could induce the cytolytic activity of T cells against tumor cells in vitro, allogeneic T cells were firstly cocultured with empty DCs (group 2), peptides-HB_100-108_/pulsed DCs (group 3), or peptides+HB_100-108_/pulsed DCs (group 4). Untreated T cells served as the negative control (group 1). After 24 hours, T cells were collected as effector cells and cocultured with PANC-1 cancer cells as target cells at a ratio of 5 : 1 (*E* : *T*). The activity of T cells against the target tumor cells was measured by the LDH cytotoxicity assays kit. As demonstrated in Figure 5, the percentage of tumor lysis in the peptides/HB_100-108_ group was higher than that in the negative control and empty DC groups. More importantly, T cells in the peptides+HB_100-108_ group significantly induced the lysis of tumor cells in comparison with the other three groups, indicating that peptides+HB_100-108_/pulsed DCs could stimulate high cytolytic activity of the T cells against PANC-1 in vitro.

## 4. Discussion

Immunotherapy based on peptides possesses numerous advantages including easy synthesis, low molecular weight, low toxicity, and the specific targeting of cancer cells. However, peptide-based anti-cancer vaccines encountered several limitations in the clinical setting due to several reasons including the following: (1) there is lack of CD4^+^ T cell help, (2) peptides are prone to degradation, (3) they may induce tolerance, (4) they induce low magnitude or transient immune response, and (5) there is dysfunction of DCs upon cancer [4, 22].

In the recent years, there has been a growing interest in cancer DC-based vaccines due to their promising results in achieving meaningful treatment responses with safety profile [23]. It has been documented that compared with synthetic short peptides (SSPs), the synthetic long peptides (SLPs) are efficiently processed and crosspresented by DCs in vivo, resulting in the priming of both CD4^+^ and CD8^+^ T cell responses [24, 25]. Several studies indicated that the direct vaccination with short peptides may lead to tolerance and anergy and promote the growth of tumor cells [26, 27]. In contrast, ex vivo-differentiated DCs loaded with short peptides could elicit potent antitumor immune response in vitro and in vivo [28, 29]. Thus, DCs appear to be a powerful carrier for the peptide-based vaccines.

As mentioned previously, the success of peptide-based vaccines mainly depends on the utilized peptides and selection of an appropriate adjuvant. Herein, it has been found that the covalent linking of adjuvant to tumor-associated antigens (TAAs) could result in the maturation and activation of DCs in vitro and induced a potent antitumor immune response in vivo compared to TAAs which they physically mixed with adjuvant [30, 31].

It is well known that Toll-like receptor (TLR) ligands have a potent activity to enhance the maturation of DCs and promote them to express proinflammatory cytokines and costimulatory molecules [32]. Later studies demonstrated that TLR ligands can efficiently increase the capacity of antigen crosspresentation in DCs [33]. HMGB1 protein has been shown to activate immune cells and induce signalling via interacting with different TLRs [34]. Thus, the addition of HMGB1 protein or its derivative peptides to the peptide-based subunit vaccines could be a promising strategy to enhance crosspresentation and crosspriming in DCs.

In this study, three antigenic synthetic peptides, which are overexpressed in several cancer types, were covalently linked with HMGB1-derived peptide (HB_100-108_) as immunoadjuvant via double R linker and loaded into immature DCs. This design includes three main advantages: (1) long synthetic peptides, (2) the covalent linking of immunoadjuvant, and (3) RR linker, which is cleaved by the protease of DCs, and thus could facilitate and enhance the processing and presentation of loaded peptides.

It is well established that only mature DCs can costimulate and activate naïve T cells and thus requiring the recognition of peptide-MHC complexes by T cell receptor and interactions between costimulatory molecules (CD40, CD80, and CD86) on the DCs and CD28 receptors on target T cells [35]. Our data showed that peptides+HB_100-108_ (but not peptides-HB_100-108_ or free HB_100-108_)/pulsed DCs exhibited high levels of several surface molecules (HLA-DR, HLA-ABC, CD83, CD80, CD86, and CD40), indicating that peptides+HB_100-108_ could efficiently trigger the maturation and activation of immature DCs. Furthermore, peptides+HB_100-108_/pulsed DCs expressed a high level of CCR7, which plays an important role in the migration of DCs to lymph nodes where they activate naïve T cells and elicit specific immune response [36].

It is well known that the secretion of inflammatory cytokines by DC-loaded antigen plays a crucial role in the induction of antigen-specific antitumor immune responses [37]. In this study, peptides+HB_100-108_ (but not peptides-HB_100-108_ or free HB_100-108_)/pulsed DCs secreted high levels of IL-6, TNF-*α*, IFN-*γ*, and IL-12p70 and low levels of IL-10. The high level and low level of IL-12p70 and IL-10, respectively, indicate that peptides+HB_100-108_/pulsed DCs could induce the differentiation of T cells toward Th2 phenotype [38].

These findings are consistent with previous studies indicating that the free HMGB-1-derived peptide has almost no effect on the maturation and activation of DCs [31]. They are also consistent with results of studies showing that the adjuvant covalently linked to the short peptides could stimulate higher epitope recognition than the physically mixed formulation of the same molecules [39, 40]. To achieve maximum immunostimulatory effect, immunoadjuvant should be in close proximity with antigen [41]. Our data revealed that peptides+HB_100-108_, but not free HB_100-108_, effectively enhanced the maturation and activation of DCs after rapid phagocytosis. The potential reason is that linking epitopes with HB_100-108_ might promote the peptide multimerization and lead to an efficient binding to the receptor of DCs via cross-linking [10]. It is important to mention that in case of direct vaccination, rather than ex vivo DC loading, peptides+HB_100-108_ will be taken up and presented by the same APCs by which can prevent the nonspecific DC stimulation. In conclusion, HB_100-108_ might exhibit a synergistic effect when covalently linked with antigenic peptides and thus could lead to maximizing the antitumor immune response.

Several studies indicated that the efficient DC vaccine should finally activate antigen-specific T cell responses. Thus, the ability of cytokine secretion is critical to confirm the activation status of T cells upon encountering the presented antigen by DCs [42]. In the present study, we found that the expression of IL-4, IL-6, TNF-*α*, and IFN-*γ* but not IL-10 dramatically increased after T cell interaction with peptides+HB_100-108_/pulsed DCs, indicating that this vaccine design has the ability to activate allogeneic T cells against tumor antigen.

The ultimate goal of cancer vaccine is to drive the activated T cells toward the tumor site and selectively kill cancer cells. In this study, T cells which were activated by (survivin, Her2, and CEA)+HB_100-108_/pulsed DCs showed a significantly high cytolytic activity against the PANC-1 cell line in vitro. This suggests that T cell stimulated by peptides+HB_100-108_-pulsed DCs could exert significant antigen-specific lysis on pancreatic cancer cells which express survivin, Her2, and CEA peptides.

In conclusion, our findings provide evidence that the covalently linked antigenic peptides with immunoadjuvant HB_100-108_ are efficiently phagocytized by DCs and induce their full maturation and activation. Those activated DCs have the ability to activate allogeneic T cells and enhance their cytolytic activity against cancer cells. This work suggests a potent strategy to improve DC-based peptide immunotherapy and supports further studies to test the efficacy of this vaccine in clinical trials.

## Data Availability

The relevant data used to support the findings of this study are included within the article.

## Supplementary Materials

Supplementary Materials Supplementary Figure 1: determination of the optimum concentration of peptides+HB_100-108_ that promote the highest level of DC maturation and activation.
