# Supplementary material for: Triggered Immune Response Induced by Antigenic Epitopes Covalently Linked with Immunoadjuvant-Pulsed Dendritic Cells as a Promising Cancer Vaccine
Source: J Immunol Res. 2020 Apr 4;2020:3965061. doi: 10.1155/2020/3965061 (PMC7160722; doi:10.1155/2020/3965061)
Supplement: Supplementary Materials — Supplementary Figure 1: determination of the optimum concentration of peptides+HB100-108 that promote the highest level of DC maturation and activation. [file 3965061.f1.pdf]

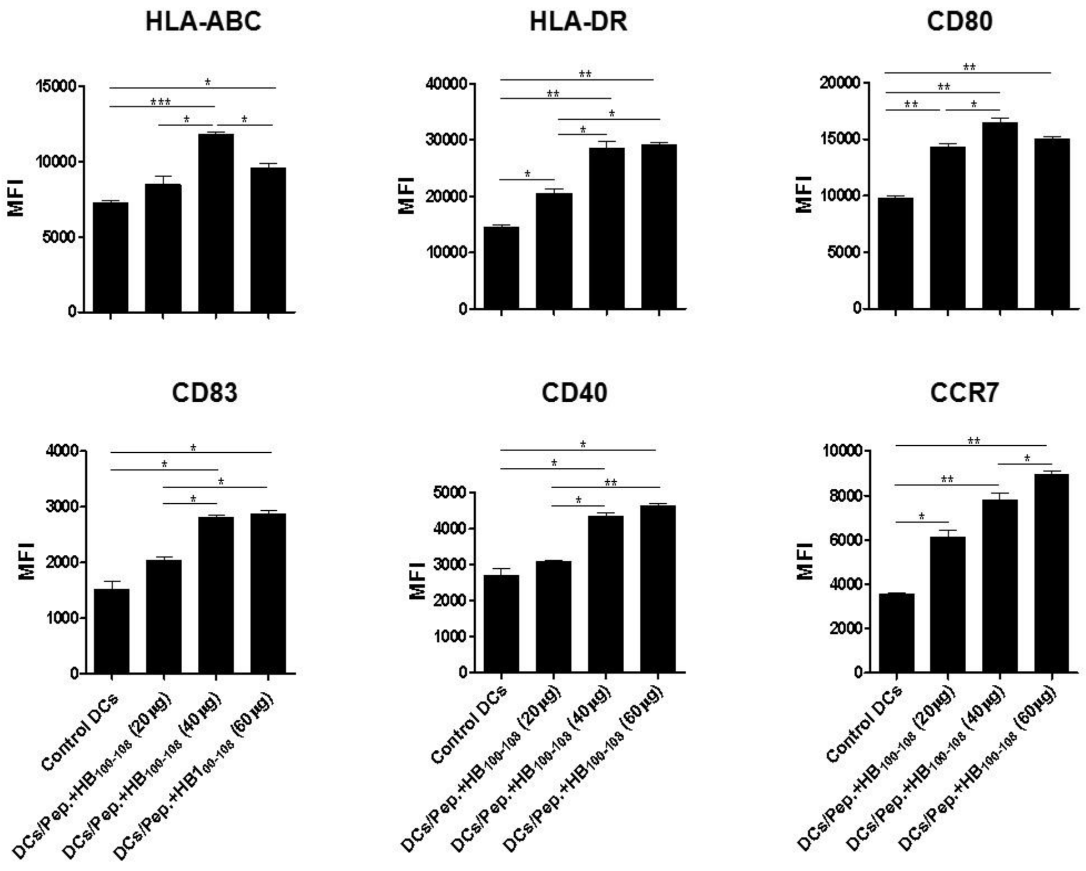

Supplementary Figure 1: Determination the optimum concentration of peptides+$HB_{100-108}$ that promote the highest level of DCs maturation and activation. Immature moDCs were left untreated or pulsed with peptides±$HB_{100-108}$ (each with 20, 40 or 60 μg/ml) for 1 hour at 37℃. Then, cells were washed and cultured overnight. The expression of HLA-ABC, HLA-DR, CD80, CD83, CD40 and CCR7 was measured by flow cytometry. Results represent the mean ± SEM of three independent experiments.
